# In vivo precision of three HR-pQCT-derived finite element models of the distal radius and tibia in postmenopausal women

**DOI:** 10.1186/s12891-016-1238-x

**Published:** 2016-09-13

**Authors:** C. E. Kawalilak, S. A. Kontulainen, M. A. Amini, J. L. Lanovaz, W. P. Olszynski, J. D. Johnston

**Affiliations:** 1 Department of Mechanical Engineering, College of Engineering, University of Saskatchewan, 57 Campus Drive, Saskatoon, SK S7N 5A9 Canada; 2 College of Kinesiology, University of Saskatchewan, Saskatoon, Canada; 3 College of Medicine, University of Saskatchewan, Saskatoon, Canada

**Keywords:** Distal radius, Distal tibia, Finite element modeling, HR-pQCT, Precision

## Abstract

**Background:**

The distal radius is the most common osteoporotic fracture site occurring in postmenopausal women. Finite element (FE) modeling is a non-invasive mathematical technique that can estimate bone strength using inputted geometry/micro-architecture and tissue material properties from computed tomographic images. Our first objective was to define and compare in vivo precision errors for three high-resolution peripheral quantitative computed tomography (HR-pQCT, XtremeCT; Scanco) based FE models of the distal radius and tibia in postmenopausal women. Our second objective was to assess the role of scan interval, scan quality, and common region on precision errors of outcomes for each FE model.

**Methods:**

Models included: single-tissue model (STM), cortical-trabecular dual-tissue model (DTM), and one scaled model using imaged bone mineral density (E-BMD). Using HR-pQCT, we scanned the distal radius and tibia of 34 postmenopausal women (74 ± 7 years), at two time points. Primary outcomes included: tissue stiffness, apparent modulus, average von Mises stress, and failure load. Precision errors (root-mean-squared coefficient of variation, CV%_RMS_) were calculated. Multivariate ANOVA was used to compare the mean of individual CV% among the 3 HR-pQCT-based FE models. Spearman correlations were used to characterize the associations between precision errors of all FE model outcomes and scan/time interval, scan quality, and common region. Significance was accepted at *P* < 0.05.

**Results:**

At the distal radius, CV%_RMS_ precision errors were <9 % (Range STM: 2.8–5.3 %; DTM: 2.9–5.4 %; E-BMD: 4.4–8.7 %). At the distal tibia, CV%_RMS_ precision errors were <6 % (Range STM: 2.7–4.8 %; DTM: 2.9–3.8 %; E-BMD: 1.8–2.5 %). At the radius, Spearman correlations indicated associations between the common region and associated precision errors of the E-BMD-derived apparent modulus (ρ = −0.392; *P* < 0.001) and von Mises stress (ρ = −0.297; *P* = 0.007).

**Conclusion:**

Results suggest that the STM and DTM are more precise for modeling apparent modulus, average von Mises stress, and failure load at the distal radius. Precision errors were comparable for all three models at the distal tibia. Results indicate that the noted differences in precision error at the distal radius were associated with the common scan region, illustrating the importance of participant repositioning within the cast and reference line placement in the scout view during the scanning process.

## Background

Osteoporosis and related fractures are global public health concerns that currently affect 200 million people worldwide [1] and are a major cause of mortality, morbidity, chronic pain, and loss of independence [2]. Distal radius fractures are the most common fracture type in postmenopausal women in North America and Europe [3–6]. Importantly, individuals who have suffered a distal radius fracture have greater risk of future osteoporotic wrist, hip, and spine fractures [7–9]. Further, 50–60 % of fragility fractures occur in women who are classified as osteopenic using dual-energy x-ray absorptiometry (DXA)-derived areal bone mineral density (aBMD)―the clinical gold standard for osteoporosis diagnosis [10–12]. Therefore, information regarding bone’s mechanical properties (e.g., bone stiffness and strength) is important in complementing fracture risk assessment beyond aBMD.

Research has shown that factors other than mineral mass or aBMD influence bone strength, such as: bone size, geometry, micro-architecture, and material properties. For instance, Kazakia and colleagues [13] have shown that two individuals can have identical DXA-derived T-scores and aBMD values but have significantly different 3D density, geometry and micro-architecture outcomes. Bone geometry and micro-architecture from HR-pQCT scans as well as strength estimates from finite element (FE) modeling have been reported to differentiate between women who have sustained a fracture relative to their non-fracture counterparts [14, 15] and identify those at high risk of fracture [16, 17]. FE modeling is an engineering technique that can be applied to non-invasively simulate mechanical testing of bone. Using bone geometry/micro-architecture and tissue material properties derived from computed tomographic (CT) images, FE modeling can estimate bone mechanical properties in vivo [18–20].

Currently, there are three main types of HR-pQCT-based FE models: 1) homogeneous single-tissue model (STM) which models the distal radius or tibia as being comprised of empty voids and bone tissue with the same material stiffness (i.e., elastic modulus, E) for both cortical and trabecular bone [18]; 2) homogeneous dual-tissue model (DTM) which separates cortical and trabecular bone (as well as voids) using different E’s for each bone tissue [21]; and 3) a scaled model which links imaged bone mineral density (BMD) with E for each voxel via density-modulus E-BMD relationships [20].

Validation studies, comparing FE modeling to mechanical testing using cadaveric forearms, demonstrated close correlations between experimental findings and FE-derived bone failure load or ultimate stress (STM: *R*^*2*^ = 0.66–0.94) [18, 22, 23] and stiffness (STM: *R*^*2*^ = 0.97; E-BMD: *R*^*2*^ = 0.98) [20]. These promising results offer great potential for HR-pQCT-based FE assessments of wrist fracture risk in populations prone to osteoporotic fractures, such as postmenopausal women. While the validity of the STM and E-BMD models have been investigated, there is little known about the repeatability (or precision) of all three FE models (i.e., STM, DTM, and E-BMD).

In order to detect and monitor small changes in bone strength over time and assess intervention/treatment effects, high measurement precision is fundamental [24, 25]. Other than the precision of the input parameters (i.e., density, geometry, and micro-architecture) and time period between repeat scans (scan interval) [26], factors that may affect precision at distal bone sites include: limb and reference line repositioning (quantified as scan common region) [27, 28], and the degree of movement artifact (scan quality) [28, 29]. To date, two precision studies report reproducibility for the STM model using cadaveric forearms [30] and young adults [31]. Cadaveric precision errors, reported as percent coefficient-of-variation (CV%), were 2.9 and 2.6 % for stiffness and failure load, respectively [30]. Short-term in vivo precision errors for women, reported as root-mean-squared coefficient-of-variation (CV%_RMS_), were 1.4 and 3.2 % for average von Mises stress at the radius and tibia, respectively [31]. Other commonly reported variables include apparent modulus, and percent load carried by the cortical and trabecular bone (*for DTM*) (Table 1) [16, 18, 20, 21, 23, 28, 30–40]; however, precision error for these outcomes is unknown. Further, it remains unknown whether outcomes of the commonly used FE models are comparably repeatable―especially in postmenopausal women, a population most in need of accurate and reliable bone strength estimates.

**Table 1:** Current literature using HR-pQCT finite element (FE) modeling for uniaxial compression simulations in the older human bone; illustrating the type of FE model used, the elastic modulus (E) used in the model, the reported outcomes, and the sites measured per study. Literature is listed in chronological order by year

Reference	FE Model^a^	Elastic modulus (E)	Outcomes reported	Site measured
Pistoia et al. Bone 2002; 30(6): 842–848.	STM	10 GPa	1. Failure Load (N)	Cadaver Radius
MacNeil et al. Med Eng and Phys 2007; 29: 1096–1105.	STM	10 GPa	1. Reaction Load (N) 2. Strain Energy Density 3. Average von Mises Stress (MPa)	Cadaver Radius (Cube Sample)
MacNeil et al. Med Eng and Phys 2008; 30: 792–799.^b^	STM	Calculated^c^	1. Elastic Modulus (CV%_RMS_)^b^ 2. Reaction Force (CV%_RMS_)^b^ 3. Average von Mises Stress (CV%_RMS_)^b^ 4. Strain Energy Density (CV%_RMS_)^b^	In vivo Radius and Tibia
MacNeil et al. Bone 2008; 42: 1203–1213.	STM E-BMD	6829 MPa E_element_ = 15004 × (ρ/1200 mg HA/cm^3^)^1.7^	1. Apparent Bone Strength (Ultimate Stress) (GPa)	In vivo Radius
Boutroy et al. JBMR 2008; 23(3): 392–399.	DTM	Cortical: 20 GPa Trabecular: 17.5 GPa	1. Stiffness (kN/mm) 2. % Load Carried by Each Tissue^d^ 3. Average von Mises Stress for each Tissue^d^ (MPa)	In vivo Radius and Tibia
Mueller et al. Bone 2009; 44: 364–371.	STM	10 GPa	1. Strength (N) 2. Stiffness (N/mm)	Cadaver Radius
Dalzell et al. Osteoporos Int 2009; 20: 1683–1694.	STM	10 GPa	1. Stiffness (N/mm) 2. Failure Load (N)	In vivo Radius and Tibia
Varga et al. J Biomech 2009; 42: 1726–1731.	DTM	Cortical: 16.5 GPa Trabecular: 2974.0 MPa	1. Failure Load (N) 2. Stiffness (N/mm)	Cadaver Radius
Burghardt et al. JBMR 2010; 25(12): 2558–2571.	DTM	Cortical: 10 GPa Trabecular: 10 GPa	1. Stiffness (N/mm) 2. Apparent Modulus (N/mm^2^) 3. Failure Load (N) 4. % Load Carried by Cortex	In vivo Radius and Tibia
Vilayphiou et al. Bone 2010; 46: 1030–1037.	DTM	Cortical: 20 GPa Trabecular: 17 GPa	1. Failure Load (N) 2. Stiffness (kN/mm) 3. % Load Carried by Each Tissue^d^ 4. Average von Mises stress for each Tissue^d^ (MPa)	In vivo Radius and Tibia
Varga et al. Bone. 2010; 47: 982–988.	STM	15 GPa	1. Stiffness (kN.mm) 2. Failure Load (kN)	Cadaver Radius
Vilayphiou et al. JBMR 2011; 26(5): 965–973.	DTM	Cortical: 20 GPa Trabecular: 17 GPa	1. Failure Load (N) 2. Stiffness (kN/mm) 3. % Load Carried by Each Tissue^d^ 4. Average von Mises stress for each Tissue^d^ (MPa)	In vivo Radius and Tibia
Macdonald et al. JBMR 2011; 26(1): 50–62.	STM	6829 MPa	1. Stiffness (N/mm) 2. Apparent Bone Strength (Ultimate Stress) (MPa) 3. Failure Load (N) 4. % Strain Energy Carried by each Tissue^d^	In vivo Radius and Tibia
Varga et al. Biomech Model Mecahnobiol 2011; 10: 431–444.	DTM	Cortical: 15 GPa Trabecular: 15 GPa	1. Stiffness (kN/mm) 2. Failure Load (kN) 3. Apparent Modulus (kN/mm^2^) 4. % Load Carried by each Tissue^d^	Cadaver Radius
Rizzoli et al. Osteoporos Int 2012; 23: 305–315.	DTM E-BMD	Cortical: 20 GPa Trabecular: 17 GPa E_element_ = 15004 × (ρ/900 mg HA/cm^3^)^1.1^	1. Failure Load (N) 2. Stiffness (kN/mm) 3. Average von Mises stress for each Tissue^d^ (MPa)	In vivo Radius and Tibia
Nishiyama et al. Osteoprosis Int 2012; 24(5): 1733–1740	STM	6829 MPa	1. Apparent Bone Strength (Ultimate Stress) (MPa) 2. % Load Carried by each Tissue^d^	In vivo Radius and Tibia
Ellouz et al. Bone 2014; 63: 147–157	DTM	Cortical: 20 GPa Trabecular: 17 GPa	1. Stiffness (kN/mm) 2. Average von Mises stress (MPa) for Each Tissue^d^ 3. % Load Carried by each Tissue^d^	In vivo Radius and Tibia

^a^ *STM* single tissue model, *DTM* dual tissue model, *E-BMD* scaled model based on bone mineral density

^b^Results of this study only report long-term and short-term precision errors (CV%), outcome values not reported

^c^Elastic modulus (E) was calculated in this study based on the reaction force required to induce 1 % strain over the average area of the slices within the section

^d^“Each tissue” refers separately to the cortical and trabecular tissues

The first objective of our study was to define and compare in vivo precision errors across three currently used HR-pQCT-based FE models (STM, DTM, scaled E-BMD) at the distal radius and tibia in postmenopausal women. The second objective was to determine the associations among time between follow-up scans (scan interval), scan quality, and common region on precision errors of all primary outcomes for each of the three FE models for the radius and tibia.

## Methods

### Participants

Measurements were completed on a sample of 34 postmenopausal women (74 ± 7 years) from the Saskatoon cohort of the Canadian Multi-centre Osteoporosis (CaM*os*) Study [41]. Postmenopausal status was assessed using a questionnaire [42]. Osteoporosis status was based on femoral neck (FN) T-scores obtained from the Saskatoon CaM*os* database; specifically, five women were osteoporotic, 20 osteopenic and nine had normal T-scores [2, 41]. Participants filled out a questionnaire regarding their medication use for 12 months prior to their baseline visit, where we recorded anti-osteoporosis medications (i.e., hormone replacement therapy and bisphosphonates) [43]. Of our participants in this precision study, no participants were using hormone replacement therapy and two participants were on bisphosphonates. Participant consent was attained prior to the study. This study was approved by the University of Saskatchewan Biomedical Research Ethics Board.

### HR-pQCT imaging

Repeat measurements were performed with an average 10 days (SD 4 days) between baseline and follow-up. As per standard protocol, all participants had their non-dominant arm and ipsilateral leg immobilized in the manufacturer-provided cast during scanning [41]. At the distal radius and tibia, a standard 9.02 mm region of interest (110 parallel CT slices) was obtained using HR-pQCT (XtremeCT; Scanco Medical AG, Brüttisellen, Switzerland) with an isotropic voxel size of 82 μm [43]. The region of interest was located 9.5 mm (radius) and 22.5 mm (tibia) proximal from the reference line placement which was positioned from the mid-region of the radial endplate and the tibial plafond, respectively [41]. The scan time was <2.8 min and the effective dose was <4 μSv per scan [43].

### HR-pQCT image analysis

One investigator (CEK) scanned, graded, and analyzed all HR-pQCT images. All images were graded for quality according to the manufacturer’s 5-point scale [41, 44]. Five radius and two tibia images with a quality of 4 or 5 were excluded from the study. In total, 27 radii and 32 tibiae were included in this current investigation and had scan qualities between 1 and 3.

Image analysis was completed according to the manufacturer’s standard evaluation and dual-threshold evaluation protocols (Scanco Module 64-bit IPL V5.08b). Briefly, standard image evaluation was used to define the periosteal surface of the radius and tibia using a semi-automatic edge-finding algorithm in a slice-by-slice manner, as described elsewhere [41, 43]. Modification of the periosteal contour line was done when it deviated from the outer bone surface. Once the standard evaluation was completed, the dual-threshold method was performed to separately define the cortical and trabecular bone tissues at both skeletal sites [45]. For the dual-threshold technique, the periosteal contour was imported from the standard evaluation image files and the endocortical contour was automatically created using a series of morphological operations (i.e., dilation and erosion) to separate the trabecular and cortical regions [45]. Modification of the endocortical contour line was done when it deviated from the endocortical surface, as previously described [46]. To separate bone from all other voxels (i.e., void, marrow, etc.), a fixed global threshold (400 mg HA/cm^3^) was applied automatically by the software during image processing.

### Finite Element (FE) modeling

All three FE models (STM, DTM, E-BMD) had linear-elastic, isotropic material properties. They were generated and solved using the Image Processing Language (IPL; version 1.15) software provided by Scanco Medical. FE models were created by converting every voxel in the scanned volume of interest (VOI) into 8-node brick elements [19, 47]. Image voxels in the VOI were converted to ~2.6 million elements at the radius and ~4.1 million elements at the tibia. Young’s moduli (E) and Poisson’s ratio are specified below. Boundary conditions were set to simulate a “high-friction” axial compression test with 1 % axial compressive strain applied to the distal surface of the bone. The high-friction nature of the simulation resulted in suppression of nodal displacement in the *x* and *y* directions at the distal surface and all directions at the proximal surface.

### Single Tissue Model (STM)

The STM is a discrete homogeneous model where all the bone voxels are assigned a single user-defined E, in this case the standard E = 10 GPa with Poisson’s ratio = 0.3 [18, 19]. Segmentation, with manual contour correction, took ~30 min per radius or tibia. Using the standard Scanco workstation, the STM solved in ~3 h per radius model and ~5 h per tibia model.

### Dual Tissue Model (DTM)

The DTM is a discrete model with cortical- and trabecular-specific E-values of 20 GPa and 17 GPa, respectively [21, 33]. Poisson’s ratio was set to 0.3 [19]. Segmentation, with manual contour correction, took ≤3 h per radius image and ≤5 h per tibia. Using the standard Scanco workstation, the DTM solved in ~3 h per radius model and ~5 h per tibia model.

### Density-based (E-BMD) model

The density-based E-BMD model is a scaled model where the E of each element was derived from the gray-value of the corresponding voxel in the image [20, 48], using density-modulus Eq. (1) proposed by MacNeil and Boyd [20]:

1 $$ E=15,004{\left(\frac{\uprho}{1200}\right)}^{1.7} $$

Where ρ is the density associated with each voxel. With this equation, individual E (MPa) are scaled in relation to fully mineralized bone (1200 mg HA/cm^3^) and the coefficients in Eq. (1) have been derived from experimental testing [20]. Poisson’s ratio was set to 0.3 [19]. The scaled E-BMD model solved in ~5 h per radius model and ~10 h per tibia model using the standard Scanco workstation.

The four primary outcomes for each model included: *bone stiffness* (kN/mm), calculated as the average reaction force at the distal surface divided by the applied displacement (0.0902 mm, corresponding to 1 % strain with a 9.02 mm thick region); *apparent modulus* (MPa), calculated as the average reaction force at the distal surface divided by estimated cross-sectional area and a fixed known strain (1 %); *average von Mises stress* (MPa); and *failure load* (kN), defined using the criterion developed by Pistoia et al. [18]. With this approach, fracture was assumed to occur when 2 % of the bone tissue exceeded a critical energy equivalent strain limit of 7000με. For DTM, a critical energy equivalent strain limit of 3500με was used because E for cortical bone (E = 20 GPa) was twice that used with Pistoia’s model (E = 10 GPa) [21]. We chose these four primary outcomes because they are the most commonly reported in the literature (Table 1) and common to all three FE models. Secondary outcomes included: the proportion of von Mises stress and the percentage of the ultimate failure load carried by the cortex and trabecular bone tissues (*DTM only*).

### Statistical analysis

We assessed the precision error of each outcome for all FE models by calculating root-mean-squared coefficients of variation (CV%_RMS_) [24, 41].

2 $$ CV{\%}_{RMS} = \sqrt{{\displaystyle {\sum}_{j=1}^m\left({\frac{\left(\frac{S{D}_j}{{\overline{x}}_j}x\ 100\%\right)}{m}}^2\right)}} $$

where *SD*_*j*_ was the sample standard deviation between the two measurements, *x*_*j*_ was the mean of the two measurements, and *m* was the number of participants in the analysis [24].

We determined the distribution of our dataset by calculating the skewness Z-score for all variables. The variables with skewness z-scores greater than 1.96 had a non-parametric distribution and were normalized using square root transformation. For Objective 1, we compared individual transformed CV% across the three FE models using multivariate analysis of variance (MANOVA) followed by pairwise comparison. MANOVA models were adjusted for multiple comparisons using Bonferroni correction. For Objective 2, we performed Spearman correlations (ρ) to determine the factors associated with the time between follow-up scans (scan interval), scan quality, and common region on precision errors of all outcomes for each of the three FE models for the radius and tibia. Significance was set to *P* < 0.05. All statistical analyses were performed using IBM SPSS commercial statistics software (PASW, Version 23 for Windows, SPSS Inc., Chicago, IL, USA).

## Results

For each of the three FE models, mean (±SD) for outcome variables and CV%_RMS_ precision are summarized in Table 2 for the distal radius and in Table 3 for the distal tibia. For the primary outcomes at the distal radius, CV%_RMS_ precision for all models were <9 % (Range STM: 2.8–5.3 %; DTM: 2.9–5.4 %; E-BMD: 4.4–8.7 %). At the distal tibia, CV%_RMS_ precision for all models were <6 % (Range STM: 2.7–4.8 %; DTM: 2.9–3.8 %; E-BMD: 1.8–2.5 %).

**Table 2:** Mean (±SD) of the baseline and follow-up scans, mean (±SD) of both measurements, root-mean-square precision error (CV%_RMS_) for stiffness, apparent stiffness, average von Mises stress, and failure load from 3 different FE models at the distal radius in postmenopausal women

Radius (*n* = 27)		First scan	± SD	Second scan	± SD	Mean	± SD	CV%_RMS_
*Single Tissue Model (STM)*								
Stiffness	*(kN/mm)*	57.2	±11.7	56.1	± 11.7	56.6	± 11.7	3.4
Apparent Modulus	*(MPa)*	1296.3	± 340.2	1257.1	± 307.7	1276.7	± 320.6	5.3
Average von Mises Stress	*(MPa)*	58.0	± 6.1	57.0	± 5.9	57.5	± 5.8	3.8
Failure Load	*(kN)*	2.944	± 0.542	2.886	± 0.543	2.915	± 0.540	2.8
*Dual Tissue Model (DTM)*								
Stiffness	*(kN/mm)*	106.8	± 22.3	105.2	± 22.3	106.0	± 22.2	3.3
Apparent Modulus	*(MPa)*	2428.5	± 652.3	2355.2	± 583.90	2391.9	± 611.6	5.4
Average von Mises Stress	*(MPa)*	108.1	± 12.2	106.2	± 11.5	107.2	± 11.5	3.8
Failure Load	*(kN)*	2.535	± 0.488	2.505	± 0.489	2.520	± 0.486	2.9
Cortical Bone								
Average von Mises Stress	*(MPa)*	153.5	± 7.9	152.2	± 7.4	152.8	± 7.5	1.5
% Minimum Load Carried	*(%)*	48.3	± 8.4	48.4	± 7.7	48.3	± 7.8	6.1
% Maximum Load Carried	*(%)*	77.6	± 9.0	77.8	± 8.9	77.7	± 8.8	2.2
Trabecular Bone								
Average von Mises Stress	*(MPa)*	76.6	± 12.1	74.4	± 11.9	75.5	± 11.4	6.7
% Minimum Load Carried	*(%)*	51.8	± 8.4	51.6	± 7.7	51.7	± 7.8	7.1
% Maximum Load Carried	*(%)*	22.4	± 9.0	22.2	± 8.9	22.3	± 8.8	9.5
*Scaled E-BMD Model*								
Stiffness	*(kN/mm)*	41.1	± 12.6	40.6	± 12.0	40.8	± 12.2	4.4
Apparent Modulus	*(MPa)*	595.2	± 215.1	583.7	± 184.1	589.4	± 196.0	8.7
Average von Mises Stress	*(MPa)*	13.2	± 4.0	13.0	± 3.4	13.1	± 3.6	7.1
Failure Load	*(kN)*	1.222	± 0.373	1.194	± 0.347	1.208	± 0.357	5.0

**Table 3:** Mean (±SD) of the baseline and follow-up scans, mean (±SD) of both measurements, root-mean-square precision error (CV%_RMS_) for stiffness, apparent stiffness, average von Mises stress, and failure load from 3 different FE models at the distal tibia in postmenopausal women

Tibia (*n* = 32)		First scan	± SD	Second scan	± SD	Mean	± SD	CV%_RMS_
*Single Tissue Model (STM)*								
Stiffness	*(kN/mm)*	172.7	± 33.7	173.1	± 34.1	172.9	± 33.7	3.7
Apparent Modulus	*(MPa)*	1896.8	± 375.2	1908.3	± 374.3	1902.5	± 372.0	4.0
Average von Mises Stress	*(MPa)*	66.6	± 5.8	66.8	± 5.4	66.7	± 5.1	4.8
Failure Load	*(kN)*	8.746	± 1.570	8.761	± 1.587	8.754	± 1.571	2.7
*Dual Tissue Model (DTM)*								
Stiffness	*(kN/mm)*	316.1	± 60.9	317.2	± 61.8	316.6	± 61.0	3.4
Apparent Modulus	*(MPa)*	3477.5	± 685.3	3500.6	± 685.3	3489.1	± 680.7	3.8
Average von Mises Stress	*(MPa)*	120.6	± 10.2	120.9	± 9.4	120.7	± 9.3	3.8
Failure Load	*(kN)*	7.256	± 1.345	7.267	± 1.360	7.261	± 1.345	2.9
Cortical Bone								
Average von Mises Stress	*(MPa)*	166.4	± 5.4	166.6	± 5.6	166.5	± 5.5	0.8
% Minimum Load Carried	*(%)*	41.5	± 9.1	41.9	± 9.7	41.7	± 9.4	3.5
% Maximum Load Carried	*(%)*	61.6	± 9.9	61.8	± 10.2	61.7	± 10.0	2.1
Trabecular Bone								
Average von Mises Stress	*(MPa)*	97.9	± 11.6	97.8	± 11.4	97.9	± 10.8	5.9
% Minimum Load Carried	*(%)*	58.5	± 9.1	58.2	± 9.7	58.3	± 9.4	2.6
% Maximum Load Carried	*(%)*	38.4	± 9.9	38.2	± 10.2	38.3	± 10.0	3.5
*Scaled E-BMD Model*								
Stiffness	*(kN/mm)*	118.7	± 27.8	118.7	± 27.5	118.8	± 27.6	2.1
Apparent Modulus	*(MPa)*	910.6	± 223.7	912.8	± 224.9	911.7	± 224.0	1.8
Average von Mises Stress	*(MPa)*	16.7	± 3.3	16.8	± 3.2	16.7	± 3.2	2.0
Failure Load	*(kN)*	3.558	± 0.802	3.556	± 0.797	3.557	± 0.797	2.5

**Fig. 1:**
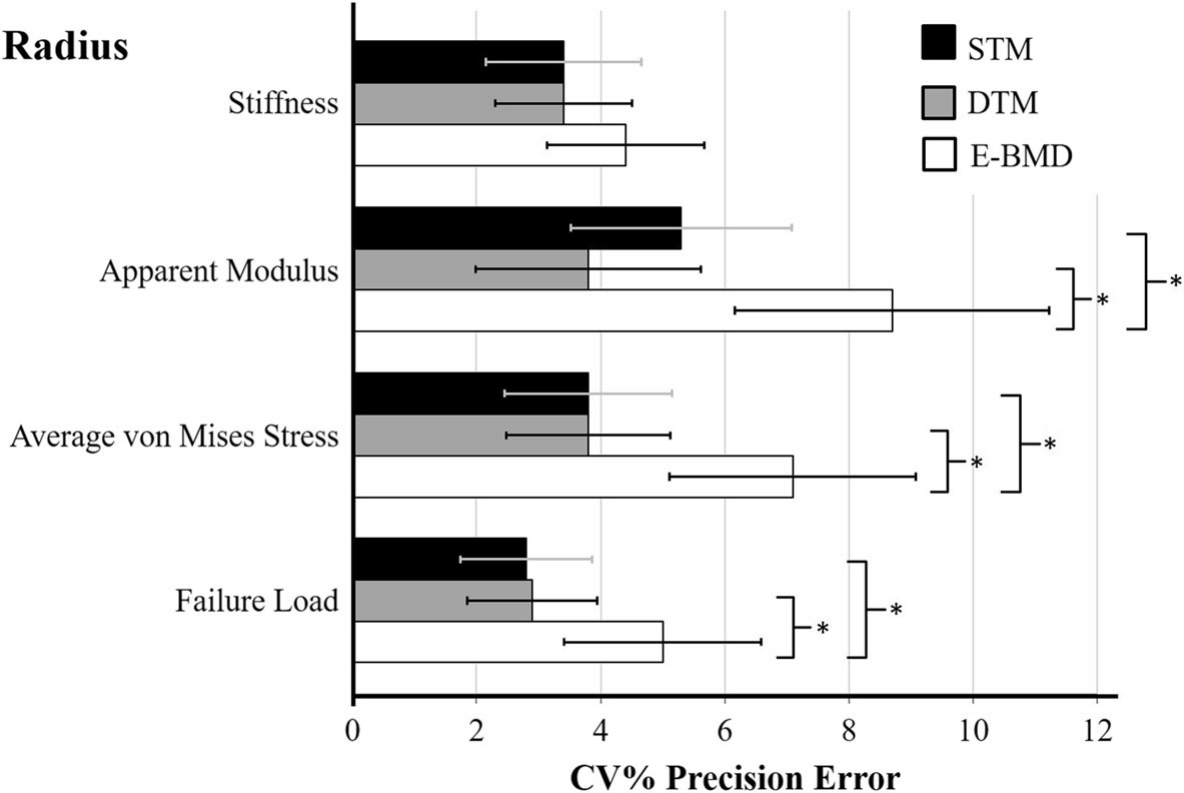
Comparison of root-mean-square precision errors (CV%_RMS_) and 95 % confidence intervals for tissue stiffness, apparent modulus, average von Mises stress, and failure load in postmenopausal women at the distal radius. * Significant at *P* < 0.05

**Fig. 2:**
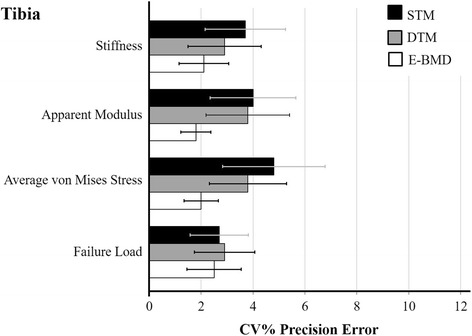
Comparison of root-mean-squared precision errors (CV%_RMS_) and 95 % confidence intervals for tissue stiffness, apparent modulus, average von Mises stress, and failure load in postmenopausal women at the distal tibia

At the radius, precision errors for the main outcomes across all three FE models were different from one another (*P <* 0.003). Precision error for apparent modulus was 3.4 % higher for the E-BMD model compared to STM (*P* = 0.015), and 3.3 % higher for the E-BMD model compared to the DTM (*P* = 0.018) (Fig. 1, Table 2). Compared to STM and DTM, the scaled E-BMD model provided 3.3 % higher precision error for average von Mises stress (*P* < 0.001) (Fig. 1, Table 2). Precision error for the failure load was 2.2 % higher for the E-BMD model compared to STM (*P* = 0.010), and 2.1 % higher for the E-BMD model compared to the DTM (*P* = 0.025) (Fig. 1, Table 2). At the distal tibia, the precision for the main outcomes across all three FE models were not different from one another (*P* = 0.156) (Fig. 2, Table 3).

There were no associations regarding the scan interval and precision errors of main FE outcomes for any of the models at the radius and tibia (*P* > 0.05). Similarly, there were no associations between scan quality and precision errors of main FE outcomes for any of the models at the radius and tibia (*P* > 0.05). The only associations were for the E-BMD model between the common scan region and the precision of apparent modulus (ρ = −0.392; *P* < 0.001) and von Mises stress (ρ = −0.297; *P* = 0.007) at the distal radius, but not the tibia (*P* > 0.05).

The common region for the distal radius was, on average, 93 % (range: 81–99 %), while at the distal tibia, the common region was 97 % (range: 88–99 %) [41]. Further, at the distal radius, there was an average slice shift of −0.8 (Range: −19.1 to 16.8 slices), whereas at the distal tibia, the average slice shift was 1.8 (Range: −6.2 to 12.8 slices).

## Discussion

The first objective of our study was to define and compare in vivo precision errors for three commonly used HR-pQCT-based FE models (two homogeneous and one scaled) at the distal radius and tibia in postmenopausal women. Precision error was significantly higher for apparent modulus, average von Mises stress, and failure load using the scaled E-BMD at the distal radius. At the distal tibia, however, all models had comparable outcomes. Reported in vivo precision errors at the radius for STM and DTM, but not E-BMD, were comparable to those reported in cadaveric forearms (CV%: Failure Load: 2.6 %, Stiffness: 2.9 % [30]) as well as previous in vivo precision at the radius in women (CV%_RMS_: Average von Mises stress: 1.4 % [31]). Our in vivo precision error at the tibia for average von Mises stress for all models was comparable to those previously reported in women (CV%_RMS_: 3.2 %, [31]).

The second objective of our study was to determine the associations regarding scan quality, and common region on precision errors of all outcomes for each of the three FE models for the radius and tibia. Results indicated that the noted differences in precision errors at the radius were significantly associated with the common scan region. It is likely that this relationship was not apparent at the tibia because of the similar common regions between the repeated measurements at this site. Previous research has suggested that even small rotation angles can lead to considerable variance in the scan region [28]. While the use of the cross-sectional area registration method within the HR-pQCT software aids in the correction of axial misplacement between baseline and follow-up scans, it does not account for possible 3D misalignments in the limb within the cast [28]. The arm cast allows for the possibility of more rotation at the wrist, while it was easier to consistently place the leg within the leg cast. The compounded effect of reference line inconsistencies between successive scans also affects the common region. This illustrates the importance of repositioning (both the limb in the cast and the reference line on the scout view).

While not an objective of the study, the actual model outcomes merit some discussion. The failure load was similar among the STM and DTM as a direct reflection of the failure criteria and boundary conditions (i.e., STM: 2 % of the bone is strained to greater than or equal to 7000με with an E of ~10 GPa; DTM: 2 % of the bone is strained to greater than or equal to 3500με with an E of ~20 GPa). However, the failure load for the E-BMD model was approximately half the failure load defined by the STM and DTM at both sites. This was primarily because, due to the density-scaled nature on how E was assigned, the final E-BMD model consisted of a large number of elements with low E and high strain, thus a lower failure load. This concept is similar to the DTM adjustment where the strain limit was lowered from 7000 to 3500με to account for increasing the E from 10 GPa to 20 GPa. Without this critical strain limit adjustment, the failure load with DTM would be twice that with STM. For this case, the opposite is true (i.e., with a critical strain limit of 7000 με and E lower than 10 GPa, the failure load is lower than STM or DTM). These results indicate a higher critical strain limit is needed when estimating failure load with E-BMD—a criterion that will require validation using experimental testing. Connected to this point, it is interesting to note that Pistoia’s original model used to develop the failure load criterion employed STM with a single E equal to 10 GPa. Because a single E was assigned to all bone elements, a critical energy equivalent strain limit of 7000με or a critical energy equivalent stress limit of 70 MPa would give identical failure loads. As such, the criterion could have been established according to stress instead of strain. In fact, when we ran the failure analysis using energy equivalent stress, failure loads with E-BMD were comparable to those with STM and DTM (*results not reported*). This was because the stress analysis focused on elements with high E, not low E. These results indicate that energy equivalent stress is an appropriate metric for estimating failure loads with HR-pQCT-based FE modeling; though, this needs to be confirmed using experimental validation testing. In addition to differences in failure load, there also appeared to be differences in: stiffness, apparent modulus, and von Mises stress among the three models at both sites. Specifically, stiffness, apparent modulus and von Mises stress with DTM were approximately double the values relative to both the STM and E-BMD. These outcomes reflect the higher user-defined E with DTM (DTM: E = 20 GPa for cortical bone; STM = 10 GPa for all bone; E-BMD ≈ 15 GPa for cortical bone scaled to 1200 mg HA/cm^3^). The stiffness and apparent modulus for the E-BMD model appeared lower relative to the STM. This was also due to the density-scaled nature of the E-BMD model which resulted in a limited number of bone elements with high E (15 GPa) and a larger number with low E; whereas, with STM all bone elements had E = 10 GPa. Specifically, in the E-BMD model the elements include void and bone in the same voxel—this will lower the density of the material within the voxel and thereby lower the apparent modulus of the element (and the stiffness of the bone). This is in contrast to the STM and DTM models that binarize the bone into two materials—bone or void—and therefore all the bone elements in the model will increase the stiffness and apparent modulus similarly between the STM and DTM. MacNeil and Boyd [20] noted that usage of E = 6.829 GPa with the STM instead of E = 10 GPa gave comparable outcomes between the STM and the E-BMD model applied in this study. We elected to use E = 10 GPa for the STM as it is the most commonly applied approach for HR-pQCT-based FE modeling of the distal radius and tibia. When applying E = 6.829 GPa with the STM (*results not reported*), stiffness outcomes were closer between the models (e.g., STM stiffness: 117.9 kN/mm; E-BMD stiffness: 118.7 kN/mm). The average von Mises stress was also lower for the E-BMD model relative to the STM and DTM. This may reflect the fact the scaled E-BMD model does not use thresholding, which has been noted to increase the partial volume effect as well as the bone area and results in a lower stress [20]. Partial volume effects may also result in less stiff elements (i.e., lower E) and a less stiff overall structure, resulting in a lower average von Mises stress.

Other than accuracy and precision, additional factors to consider when deciding what model to use include: computational time, analysis time and assumptions used within the models’ (e.g., user-defined E). The STM has the shortest total analysis time (i.e., image segmentation and FE analysis) per model for both the radius (3.5 h) and tibia (5.5 h). Overall, the DTM and the E-BMD models have approximately equal analysis times at both sites (radius: 5 h; tibia: 9–10 h) because of the time commitment for dual thresholding of the cortex for the DTM. However, the E-BMD model does not require invested manual time in the segmentation part of the analysis because it uses the unsegmented files so the majority of the analysis time comes from the complex FE modeling. According to Burghardt et al. [39], the use of binarized models (i.e., STM and DTM) will facilitate understanding pertaining to how bone micro-architecture changes in response to treatment or intervention and the mechanics of bone strength. However, using the STM or DTM will not provide information regarding how changes in bone mineral affect bone strength [39] (bone mineral mass being an important factor underpinning bone strength [49]), an advantage of the E-BMD model. Therefore, depending on the research question as well as balancing precision, computational time, costs associated with analysis/segmentation, and the assumptions used within the models’, either the STM or scaled E-BMD model may be the favorable model to implement in future research.

Our study design and study cohort had strengths that warrant consideration. First, repeat measurements were separated by an average of 1 week—an important condition because underestimation of precision error has been reported when precision is calculated using scans repeated on the same day [26]. Second, our study contained enough repeat scans (*N* ≥ 27) to provide the recommended 27 degrees of freedom necessary to establish reliable precision errors with an upper 90 % confidence limit less than 30 % (e.g., if the precision error was 2 %, then we were 90 % confident that the true precision error is less than 2.6 %) [24, 50]. Third, the reported bone health status of our sample of postmenopausal women was comparable to their peers in North America, Europe, Australia and Japan [10, 41, 51], thereby suggesting our data is generalizable to postmenopausal women in these regions.

Limitations associated with this study pertain to our study sample population and sample size. First, although we studied older postmenopausal women, skeletal precision may vary according to duration or time from menopause and osteoporosis status which was not considered in this study—this merits further investigation into population-specific precision studies. A second limitation pertained to our sample size. While our sample size was sufficient to obtain the required degrees of freedom for accurate precision error estimates, it was limited to assess the possible role of osteoporosis or bone status as well as the influence of FE input parameters (i.e., density, geometry, and micro-architecture) in the comparisons.

Future studies in this area should include further investigations into the factors that affect precision error and metrics for monitoring change for each FE model. The scan quality in our study was very homogeneous and thereby did not affect the precision of our FE models; consequently, this restricted our ability to fully address the impact of these factors on precision error. Previous work has shown that poor scan quality corresponds to significantly higher precision error in outcomes [44]. Considering the densitometric, geometric, and micro-architectural information from HR-pQCT are used as input parameters for our FE models it is logical poor precision in these outcomes could lead to poor precision of FE outcomes. Future research, with a larger sample size, is needed to address this question. Further, future studies should investigate the extent to which each model can detect change in bone strength metrics and what time interval between follow-up scans are required to capture these changes.

## Conclusions

In summary, all methods provided similar precision for modeling the distal tibia, whereas the STM and DTM appeared more precise for modeling the distal radius. However, considering precision, computational time, costs associated with analysis/segmentation, and model assumptions, the scaled E-BMD model may be the favorable model to implement in future research. Noted differences in precision error at the distal radius were associated with the common scan region between follow-up measures. Study results illustrate the importance of appropriate participant (re)positioning within the cast and reference line placement in the scout view during the scanning process.

## Abbreviations

aBMD: Areal bone mineral density
CT: Computed tomography
CV%: Coefficient of variation (in percent)
CV%_RMS_: Root-means-squared coefficient of variation (in percent)
DTM: Dual tissue model
DXA: Dual-energy x-ray absorptiometry
E-BMD: *A scaled finite element model which links imaged bone mineral density (BMD) with Young’s Modulus (E) for each voxel* via *density-modulus E-BMD relationships*
FE: Finite element
FN: Femoral neck
GPa: Giga pascals
HA: Hydroxyapatite
HR-pQCT: High-resolution peripheral quantitative computed tomography
MANOVA: Multivariate analysis of variance
MPa: Megapascals
STM: Single tissue model
VOI: Volume of interest
με: Microstrain
ρ: Density
